# Yellow Fever

**Published:** 1888-08

**Authors:** 


### YELLOW FEVER.

The reports concerning the appearance of yellow fever in Matamoras are quite conflicting, as they always are whenever the disease makes its appearance at any port. Where there is smoke there is generally fire, and prudence dictates that it is best to take the benefit of the doubt, and be on guard. So far as prudence, and eternal vigilence, backed by intelligence and plenty of funds, will avail, the people of Texas may feel secure that should the disease prove to become epidemic at Matamoras, it will not find entrance into Texas *via* Brownsville and Brazos Santiago; for the faithful watchman, Dr. Wolff, is on deck at that point. State Health Officer Rutherford, too, it is understood, is practicing commendable caution, and guarding all points with vigilance. Dr. Wolff officially announces that Brownsville and Brazos Santiago are free from infectious disease, and in good sanitary condition

Our people are learning, by experience, at last, that "prevention" is not only "better than cure," with reference to epidemic diseases, but also much cheaper. The JOURNAL, some time ago, gave the details of a valuable lesson on this subject, which had been taught our legislators. A few years ago, in that spirit of penny-wisdom and mistaken notions of economy and retrenchment which has characterized certain Solons in Texas Legislatures, the appropriation for quarantine purposes was reduced by taking off *one guard* at a point on the Brazos. The State saved, thereby, nine hundred dollars. Yellow fever crept in at that point, and a little epidemic was lighted up, which cost $22,000 to suppress. It is to be hoped that the "Bells of Cooke," in future sessions of the Legislature, will be suppressed, and that the quarantine fund may never be restricted again to a point whereby the safety of the people will be endangered.

There is still time before frost for the introduction and development of the disease into Texas, and for an epidemic to occur; and it is to be hoped that our health authorities will relax nothing of their efforts. Florida is some distance from Texas, but with the intimate communication by rail, the appearance of yellow fever in Jacksonville, Key West or Tampa is as menacing to Texas as if it were at Matamoras.

New Orleans is declared uninfected, and is recognized by all health authorities as a clean city; and in her vigilant health authorities Texas has a friend and ally. There is little danger, we apprehend, that such a trick will be played upon them or upon State Health Officer Rutherford as that perpetrated recently, whereby five persons from the infected districts of Florida were smuggled into Tennessee. Eternal vigilance is the price of public safety.

